# Successful treatment of *Chlamydophila pneumoniae *acute respiratory distress syndrome with extracorporeal membrane oxygenator: a case report and diagnostic review

**DOI:** 10.1186/1752-1947-6-20

**Published:** 2012-01-17

**Authors:** David De Bels, Philippe Gottignies, Marijke Reynders, Sébastien Roques, Stephan Wilmin, Véronique-Yvette Miendje Deyi, Sophie Jamart, Jacques Devriendt

**Affiliations:** 1 Intensive Care Department, Brugmann University Hospital, 4 Place Van Gehuchten, 1020, Brussels, Belgium; 2 Microbiology Laboratory, AZ Sint Jan Ziekenhuis, 10 Ruddershove, 8000 Brugge, Belgium; 3 Microbiology Laboratory, Brugmann University Hospital, 4 Place Van Gehuchten, 1020, Brussels, Belgium

## Abstract

**Introduction:**

*Chlamydophila pneumoniae *is a respiratory pathogen known to infect the upper and lower respiratory tracts. Infection severity can range from sub-clinical pulmonary infection to acute respiratory distress syndrome.

**Case presentation:**

A previously healthy 62-year-old Caucasian man was admitted to our hospital for acute respiratory failure. Serum samples obtained every week starting from the day of admission showed clear-cut seroconversion for *C. pneumoniae *antibodies. All other cultures obtained during the first days of hospitalization were negative. Despite maximal ventilatory support (high positive end expiratory pressure, fraction of inspired oxygen of 1.0, nitric oxide inhalation, neuromuscular blocking agents and prone positioning), our patient remained severely hypoxemic, which led us to initiate an extracorporeal membrane oxygenation treatment. Extracorporeal membrane oxygenation and hemodiafiltration were withdrawn on day 12. Our patient was extubated on day 18 and discharged from our Intensive Care Unit on day 20. He went home a month later.

**Conclusion:**

We describe the first published case of acute respiratory distress syndrome due to *C. pneumoniae *infection successfully treated by extracorporeal membrane oxygenation, a very useful tool in this syndrome. A quick and specific method for the definite diagnosis of *Chlamydophila *infection should be developed.

## Introduction

*Chlamydophila pneumoniae *is an obligate intracellular Gram-negative bacterium. The spectrum of disease, in addition to pneumonia and influenza-like illness, includes pharyngitis, sinusitis, bronchitis, exacerbation of chronic obstructive pulmonary diseases and reactive arthritis [1-5]. *C. pneumonia*e accounts for 6% to 20% of cases of community-acquired pneumonia (CAP) [1,2]. Many of these cases have few symptoms and don't require hospitalization ('walking pneumonia'). However, more severe cases may occur, with up to 18% requiring hospitalization [6] and even mechanical ventilation, especially in elderly, immunocompromised hosts and patients with coexisting cardiopulmonary disease [7], but also, rarely, in previously healthy adults [8].

Old and new macrolides are effective against *C. pneumoniae *and have been recommended as first-line treatment. New fluoroquinolones are also effective *in vitro *against *C. pneumoniae *and can be used. Studies have shown that 35% to 47% of *C. pneumoniae *pneumonia is mixed with other pathogens, the most common being *Streptococcus pneumoniae *[9,10].

We describe the case of severe CAP due to *C. pneumoniae *infection in a previously healthy adult patient, with acute respiratory distress syndrome (ARDS) necessitating extracorporeal membrane oxygenation (ECMO).

### Case presentation

A previously healthy 62-year-old Caucasian man was admitted to our hospital for acute respiratory failure. Our patient developed a fever of up to 40°C seven days earlier and a non-productive cough three days later. He had not received any antimicrobial treatment prior to his hospitalization, the diagnosis of his primary care physician being influenza (A/H1N1v), given the ongoing outbreak.

His medical history was remarkable for possible viral pericarditis without any consequence in 2007 and a gastric ulcer 30 years earlier. He had no drinking habits. He did not smoke cigarettes. He had not travelled abroad recently. He did not have any bird or pet.

**Figure 1:**
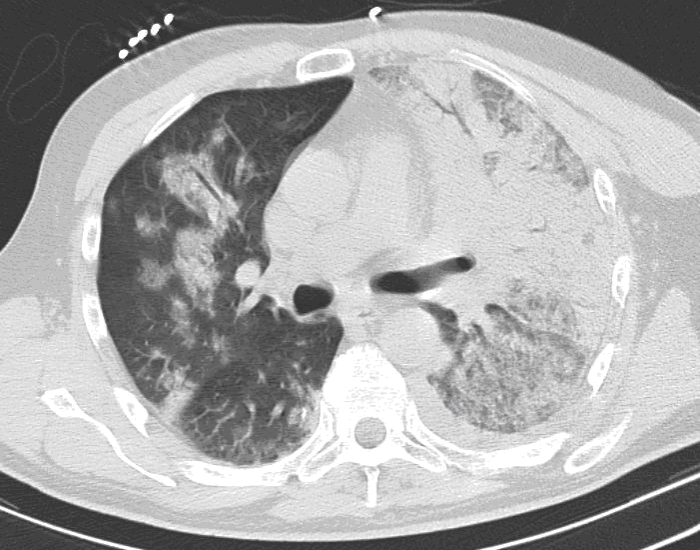
**Unenhanced computed tomography scan through the thorax showing alveolar type infiltrates of the two lung field with air bronchograms**.

On hospital admission, our patient was in acute respiratory distress. His respiratory rate was 40 breaths/minute, his temperature 38.3°C, his pulse 98 beats/minute and his blood pressure 114/60 mmHg. Auscultation revealed crackles over his whole left lung and over his right lower lung field. A computed tomography scan showed diffuse alveolar type infiltrates in his two lung fields with air bronchograms (Figure 1).

Arterial blood gas analysis (under 100% oxygen through a non-rebreathing mask) showed pH 7.54, a partial pressure of carbon dioxide (PaCO_2_) 44 mmHg, partial pressure of oxygen (PaO_2_) 38 mmHg and an arterial blood oxygen saturation of 84%. His white blood cell count was 5780 cells/μL (86% neutrophils) and the erythrocyte sedimentation rate was 92 mm/h. Laboratory values showed serum creatinine at 1.7 mg/dL, potassium at 2.8 mEq/L, creatine phosphokinase at 644 IU/L, liver test alterations (alanine transaminase at 87 IU/L), lactate dehydrogenase elevation (1708 IU/L) and D-Dimers at 7420 ng/mL, activated partial thromboplastin time of 72 seconds, normal international normalized ratio and blood platelets at 166.000/μL. His urine output was 0.4 mL/kg/h over six hours. An electrocardiogram showed a sinus tachycardia with a complete right bundle branch block.

Serum samples obtained every week as from the day of admission showed a clear-cut seroconversion for *C. pneumoniae *antibodies (the course of the antibody titers shown in Table 1).

**Table 1:** Comparison of antibody kinetics using different techniques

Test (units)	Day one	Day 13	Day 23
MIF-IgG^a ^(titer)	1/512	1/2048	1/4096
MIF-IgM^b^(titer)	< 1/10	< 1/10	< 1/10
MOMP-IgG^c ^(AU/mL)	102	478	> 500
LPS-IgG^d ^(index)	1.5	6.8	9.4
LPS-IgA^e ^(index)	< 0.9	2.8	1.3

^a^MIF-IgG (Focus Diagnostics): + when > 1/512 or a four-fold rise in titer in consecutive sera; ^b^MIF-IgM (Focus Diagnostics): < 1/16 = negative; ^c^MOMP-IgG (*C. pneumoniae *IgG-ELISA plus medac): + when ≥ 28 AU/mL; ^d^LPS-IgG (*C. pneumoniae *IgG- rELISA medac): + when index ≥ 1,1; ^e^LPS-IgA (*C. pneumoniae *IgA- rELISA medac): + when index ≥ 1,5; doubtful: index 0.9-1.4.

Paired serum samples and antigens against the most common microorganisms, including atypical bacteria and common viruses such as A/H1N1v, were negative. Blood, sputum and urine tests for bacterial cultures obtained during the first day of hospitalization were negative.

Our patient was treated by amoxycillin-clavulanic acid, moxifloxacin and oseltamivir. His respiratory status necessitated endotracheal intubation and mechanical ventilation. Severe arterial hypotension prompted norepinephrine infusion and the insertion of a pulmonary artery catheter. The initial hemodynamic pattern was typical for sepsis (hemodynamic values are shown in Table 2). Metabolic data showed a mixed venous saturation of 56%.

**Table 2:** Hemodynamic values throughout Intensive Care Unit stay till ECMO removal.

Parameters	Day one	Day five	Day nine	Day 12
HR	97	88	92	104
MAP	60	67	69	91
PCWP	18	12	7	6
CI (3.0-5.0)	3.9	4.2	2.7	4.6
ISVR (1200-2000)	861	1416	2414	1512
Noradrenaline (μg/kg/min)	1.1	0.6	0.2	0
Dobutamine (μg/kg/min)	15	20	5	0

HR: heart rate (beats per min); MAP: mean arterial pressure (mmHg); PCWP: pulmonary capillary wedge pressure (cmH_2_0); CI: cardiac index (L/min/m^2^); indexed systemic vascular resistance (Dyne.sec.m^2^/cm^5^).

Despite maximal ventilatory support (high positive end expiratory pressure, an inspired oxygen fraction (FiO_2_) of 1.0, nitric oxide inhalation of 20 ppm, neuromuscular blocking agents and prone positioning), our patient remained severely hypoxemic (PaO_2_/FiO_2 _= 38) which led us to initiate ECMO treatment.

Venovenous ECMO (a Sorin Revolution centrifugal pump, a Sorin ECCO oxygenator and a Sorin Satcrit console from Sorin Group, Milano, Italy) was put in place on the fifth day of hospitalization, with a left femoral 22-Fr drainage cannula and a right femoral 23-Fr return cannula, inducing a drastic improvement of our patient's oxygenation parameters (PaO_2 _= 120 mmHg). The mixed venous oxygen saturation (SVO_2_) increased from 56% to 86%. Continuous veno-venous hemodiafiltration (CVVHDF) renal replacement therapy was also initiated on day three because of acute renal failure.

There were no severe complications of the ECMO treatment except for hemorrhagic suffusion of the two femoral catheter insertion points, requiring a blood transfusion.

ECMO and CVVHDF were withdrawn on day 12. Our patient was extubated on day 18 and discharged from our Intensive Care Unit on day 20. He went home a month later. He is now in good physical condition and has returned to work and to a normal social life.

## Discussion

This case illustrates the polymorphism in the presentation of *C. pneumoniae *infection, which can cause severe CAP complicated by ARDS, even in immunocompetent patients. Pneumonia and bronchitis are the most common clinical infections associated with *C. pneumoniae*. The classical pulmonary presentation is a single subsegmental infiltrate, even though lobar consolidation or bilateral infiltrates can also be seen.

Two major aspects are discussed below: the rationale for the use of ECMO in ARDS patients as well as its different techniques, and the difficulties of diagnosing *C. pneumoniae *pneumonia.

### ECMO and CO_2 _removal

There are two main ECMO techniques according to the type of vascular access that is used: venovenous and venoarterial. Each one has a specific indication. Venoarterial ECMO gives cardiac and respiratory support. Indications for venoarterial ECMO include postcardiac surgery (heart surgery or heart transplantation), cardiogenic shock due to acute myocardial infarction or acute myocarditis and intoxication. For cardiac support, the goal is to optimize organ perfusion (by obtaining a SVO_2 _greater than 70%, which usually needs an output index of about 3 L/min/m^2^). This is achieved by choosing the appropriate sizes of cannula according to the patient's body surface area.

In isolated respiratory failure, a venovenous access is preferred. The objective is CO_2 _removal at least equal to the patient's metabolism (roughly 3 cm^3^/kg/min in adults). The purpose of venovenous ECMO use in ARDS patients is lung protection (reducing ventilator-induced lung injury) through a decrease in alveolar distention permitted by the reduction in ventilator conditions.

Even though a lot of progress has been done in technical issues, the risk-benefit ratio must be taken into account when ECMO is proposed to a patient with severe respiratory failure. As in all extracorporeal devices, anticoagulation is mandatory. Many patients will experience bleeding, which can be very severe. Contraindication for anticoagulation remains the most important limitation of this technique. Previous severe disability or poor prognosis due to underlying disease constitutes the other main contraindication for ECMO initiation. The main complications are bleeding, thromboembolism, cannula-related complications, pulmonary embolism or infarction, aortic thrombosis and coronary or cerebral hypoxemia; the latter three being more frequent in a venoarterial montage type.

Discussion still stands in the literature as to whether mechanical ventilation of more than seven days is or is not a relative contraindication for ECMO because of ventilation-induced lung injury [11].

The first randomized clinical trials failed to demonstrate beneficial effect of ECMO for severe respiratory failure in the 1970s and 1980s. Since then, technical improvements for ECMO on one hand, and better treatment of the ARDS (protective ventilation and others) on the other hand, have renewed interest in ECMO [12]. The recent CESAR trial (Conventional Ventilation or ECMO for Severer Adult Respiratory Failure [13]) demonstrated a possible beneficial effect of ECMO. Of those patients referred for ECMO, there was 63% survival rate at six months without disability, compared to 47% in those who were assigned to conventional management. This translates to one extra survivor without disability for every six patients treated.

A recent Italian experience, in patients with ARDS due to the influenza A/H1N1 virus, based on pre-emptive patient centralization showed a 77% survival rate if ECMO was started within seven days of initiation of mechanical ventilation [11]. Indication for ECMO in this study was refractory hypoxia or an oxygenation index below 30 despite a PaO_2_/FiO_2 _ratio greater than 100 mmHg.

Treatment of critically ill patients affected by the 2009 Influenza A (H1N1v) outbreak in Australia and New Zealand [14] included ECMO, with a 71% survival rate at Intensive Care Unit discharge, an excellent result. These three studies emphasize the renewed place of ECMO in the treatment of severe ARDS with very good survival rates, considering the severity of the initial insult.

The limitation of plateau airway pressures and the low tidal volume used in ARDS patients have been at the cost of an increased PaCO_2_. There has therefore been an increase in interest for extracorporeal CO_2 _removal techniques. Different techniques have been developed but most of them have not gone to relevant, sufficiently powered clinical trials due to technical problems or insufficient CO_2 _removal or oxygenation [15]. The pumpless extracorporeal lung assist (PECLA), or Novalung^® ^[Novalung GmbH, Heilbronn, Germany], is a compact pumpless device driven by the pressure gradient between arterial and venous blood. A femoro-femoral setting is most commonly used. The main advantage of a pumpless device is a reduction in mechanical blood trauma, bleeding and hemolysis. The Novalung^® ^has been widely studied in ARDS. Three studies have demonstrated the clinical efficacy of this PECLA device [16-18]. CO_2 _removal is efficient but oxygenation may be insufficient in these critically ill patients.

### C. pneumoniae diagnosis

Diagnosis of *C. pneumoniae *pneumonia is still debated in the current literature. According to Grayston *et al. *and Saikku *et al. *[19,20], who first described this Chlamydia species, almost everybody is infected and reinfected with *C. pneumoniae *throughout his or her life. Unspecific symptoms of *C. pneumoniae *infections make the diagnosis even more difficult, with a possible underestimation of its frequency. Undetected infections may lead to chronic disease with serious consequences, such as atherosclerotic cardiovascular disease [21] or asthma. Its incidence in CAP ranges from 3% to 22%, varying according to the diagnostic method used [22]. The most frequent routinely used diagnostic techniques are serological, including complement fixation test, immunofluorescence assay, microimmunofluorescence (MIF) and genus- and species-specific enzyme-linked immunosorbent assay (ELISA) systems. No currently available serologic tests of a single serum specimen will provide conclusive evidence of a current infection with *C. pneumoniae*.

Since the publication of the Centre for Disease Control (CDC) 2001 recommendations for Nucleic Acid Amplification Tests (NAATs) [23], a multitude of in-house polymerase chain reaction (PCR) tests have been described, though very few of them have been validated by the CDC. Results of NAATs may be unreliable because of cross-contamination, inappropriate treatment of the clinical samples (leading to the loss of the target nucleic acid) or the presence of inhibiting substances [24]. Additionally, validation of these tests is primarily analytical and not against clinically obtained specimens.

Bacterial culture has traditionally been considered the 'gold standard' for diagnosis; however, its sensitivity, even in excellent laboratories, seldom exceeds 90% and is typically between 75% and 85%. The culture is technically difficult to implement and is only available in a few laboratories worldwide [25].

Even though MIF is still actually considered as the 'serological gold standard' technique for the detection of species-specific antibodies, there is a large discrepancy between MIF testing for *C. pneumonia *and detection of these organisms by culture or PCR. The MIF test is reliable for detection of a prior exposure to Chlamydiae by the presence of immunoglobulin G (IgG) antibodies and is relatively sensitive for the detection of IgM. IgM is an unreliable marker of acute infection in adolescents and adults since it is often not present, presumably because of previous infection by a chlamydial species [24]. Additionally, MIF is quite a laborious and subjective technique that requires much experience, is not standardized and has significant laboratory-to-laboratory variations.

In our institution, in order to get better sensitivity (for example, after reinfections), better reproducibility, greater objectivity and less cross-reactivity in the serology tests for Chlamydiaceae, two-level serological testing has been implemented for *C. pneumoniae*. The first level test is a search by ELISA-technique for anti-major outer membrane protein (MOMP) IgG and IgA antibodies (*C. pneumoniae*-IgG & IgA-ELISA *plus*, Medac, Hamburg, Germany) which are species-specific. This permits us to screen patients. The disadvantage of anti-MOMP antibodies is their late appearance in acute primary infection (three weeks for IgA, six to eight weeks for IgG) and their considerable persistence in human serum afterwards, independent of the bacterial eradication. In general, the prevalence of *C. pneumoniae *IgG antibody reaches 50% in adults above 20 years of age, and 80% in the elderly population (> 70 years old). The second level test is another ELISA assay, rELISA (Medac), for the detection of genus-specific anti-lipopolysaccharide(LPS) antibodies. Chlamydiae contain, as a common immunodominant antigen, the LPS to which the first immune reaction is directed. These anti-LPSs have the main advantage of rising quickly during acute infection (five to ten days for IgA), allowing early diagnosis, and returning to normal a few weeks after the infection and are thus associated to the eradication of the bacterium [26]. The use of paired sera enables the discrimination of current infections, reinfections and reactivations (defined by titer increase) from chronic persistent ones (constant LPS, IgG and IgA antibody titers, present in 5% to 10% of the adult population) for which no antibacterial treatment is needed. The latter is due to the chronic presence of bacteria in the organism in a latent phase (monocytes). Criteria for acute infections are a four-fold increase in IgA or IgG or a doubling of IgA and IgG at 10 to 15 days.

Moreover, the described consecutive sera of our patient underwent an additional retrospective examination by a MIF assay (Chlamydia MIF IgG & Chlamydia MIF IgM, Focus Diagnostics, Cypress, CA, USA). A straightforward IgG seroconversion could also be observed (Table 1). Thanks to the detection of IgA and of IgG antibodies to *C. pneumoniae *in various combinations, serology allows a classification of the state of infection. In our patient, reinfection was diagnosed because MIF were positive on admission; there was a four-fold increase in anti-MOMP IgG and, finally, an important rise and a rapid decline in anti-LPS IgA.

## Conclusion

*C. pneumoniae *can induce very severe ARDS. We describe the first published case of ARDS due to *C. pneumoniae *infection successfully treated by ECMO. Definite *Chlamydophila *diagnosis remains a challenge but should be sought for in severe ARDS patients without evidence of other infectious cause. A quick and specific method for the definite diagnosis of *Chlamydophila *infections should be developed.

### Consent

Written informed consent was obtained from the patient for publication of this case report and any accompanying images. A copy of the written consent is available for review by the Editor-in-Chief of this journal.

## Competing interests

The authors declare that they have no competing interests.

## Authors' contributions

DDB, JD and PG have taken part in the conception, the design and the writing of the article. JD and SJ have acquired the data. SW has analyzed and interpreted the data. VYMD and MR have done all the microbiological analyses. JD, MR and SR have critically revisited it for important intellectual content. All authors read and approved the final manuscript.
